# Supplementary material for: The RNA sensor MDA5 detects SARS-CoV-2 infection
Source: Sci Rep. 2021 Jul 1;11:13638. doi: 10.1038/s41598-021-92940-3 (PMC8249624; doi:10.1038/s41598-021-92940-3)
Supplement: Supplementary file 1 — Supplementary Figures. [file 41598_2021_92940_MOESM1_ESM.pdf]

Supplementary Information

The RNA sensor MDA5 detects SARS-CoV-2 infection

Natalia G. Sampaio, Lise Chauveau, Jonny Hertzog, Anne Bridgeman, Gerissa Fowler, Jurgen P. Moonen, Maeva Dupont, Rebecca A. Russell, Marko Noerenberg, Jan Rehwinkel

Content:

Supplementary Figure 1

Supplementary Figure 2

Supplementary Figure 3

Supplementary Figure 4

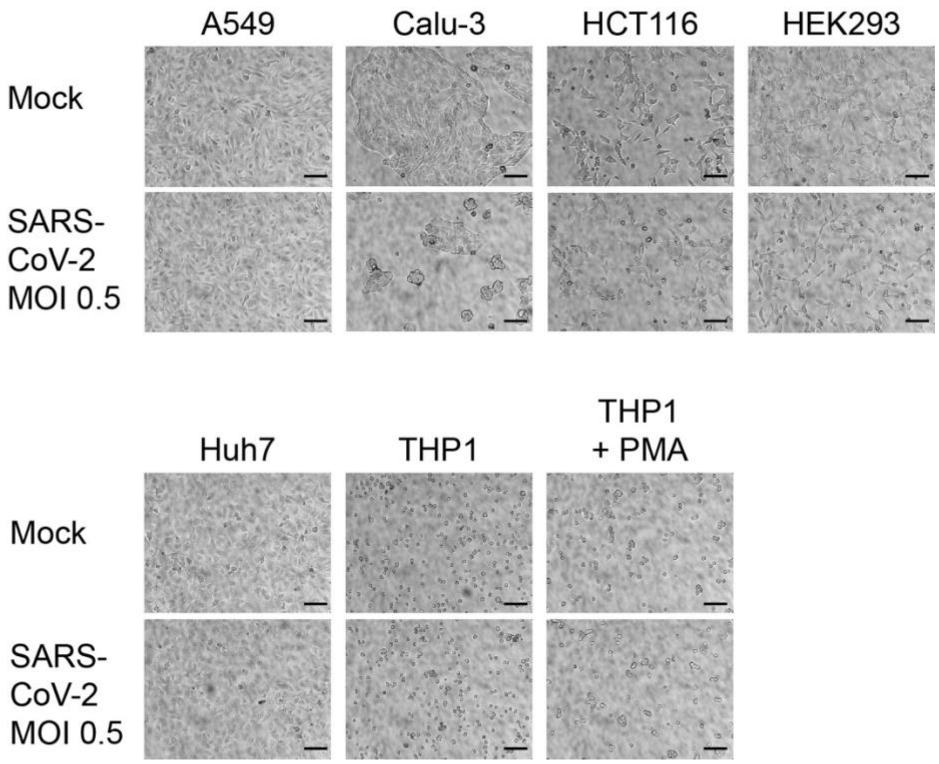

Supplementary Figure 1. Microscopy images of SARS-CoV-2 infected cells.

The indicated cell lines were mock-infected or infected with SARS-CoV-2 (MOI = 0.5) for 24 hours prior to analysis by brightfield microscopy. Scale bars represent 100 μm. Data are from a single experiment.

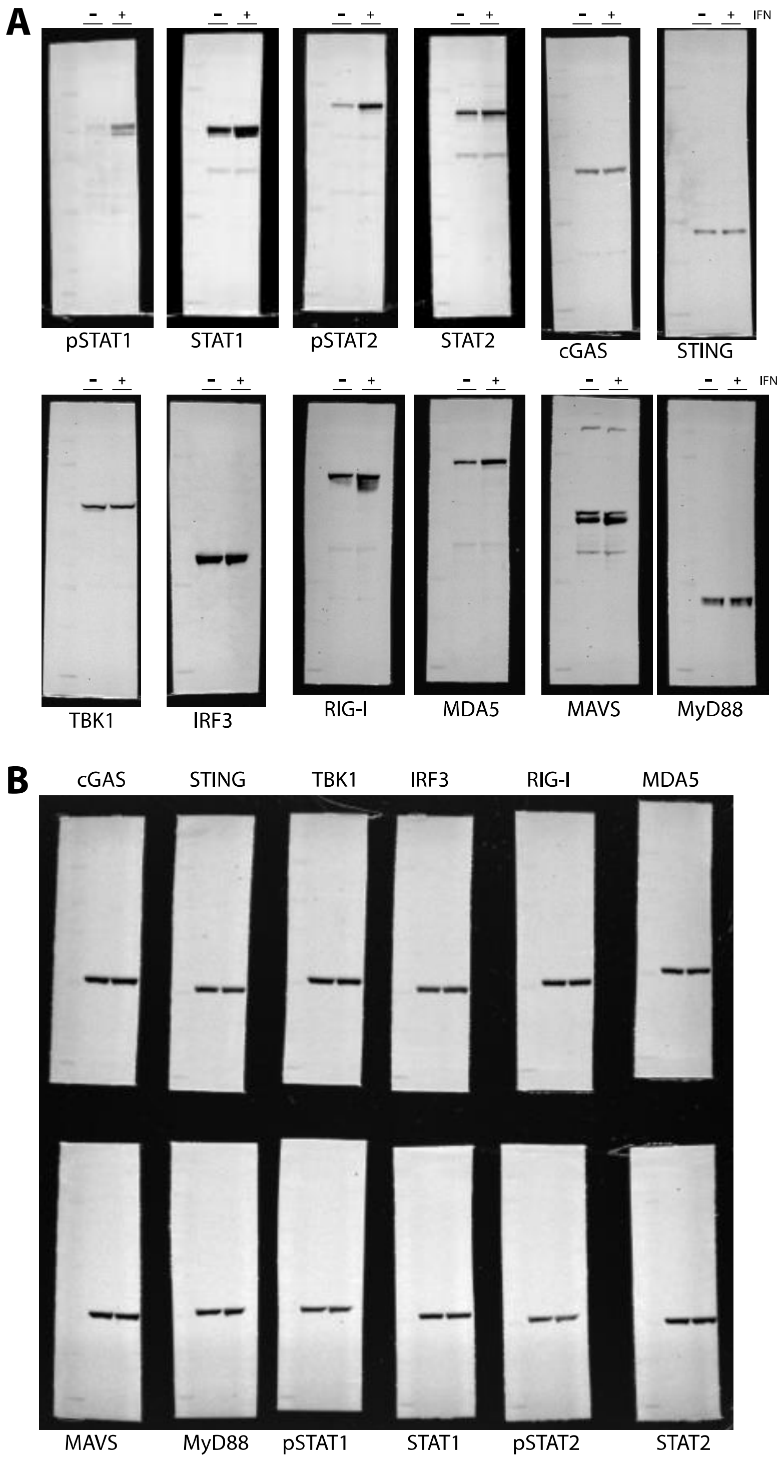

Supplementary Figure 2. Full western blots; corresponding to Figure 1B.

(A) Overlay of membranes and chemiluminescence signal for antibodies indicated below individual panels. The first lane on each membrane contains the size marker.

(B) Overlay of membranes and chemiluminescence signal for β-actin obtained after re-probing corresponding membranes from (A).

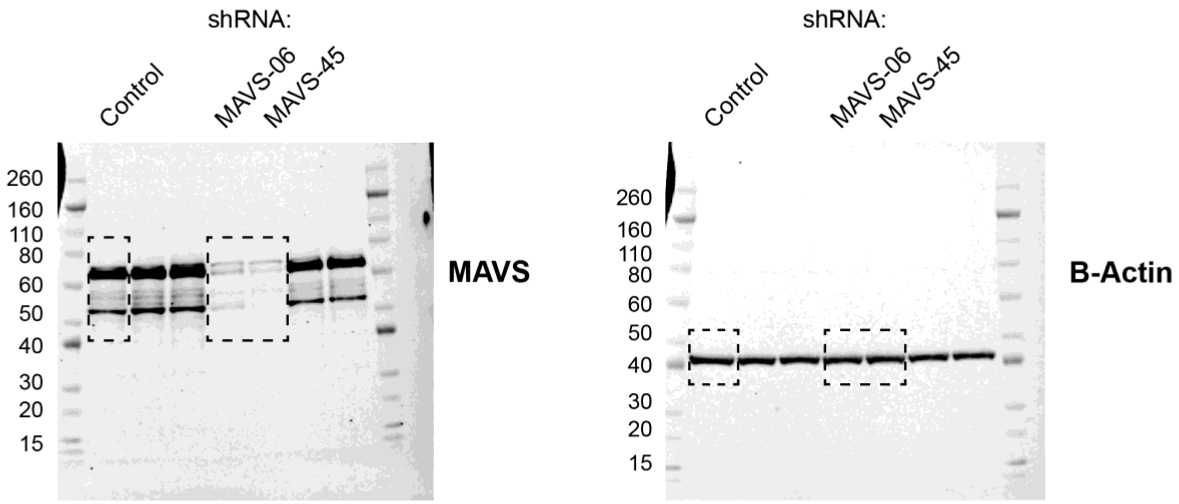

Supplementary Figure 3. Full western blots; corresponding to Figure 2A.

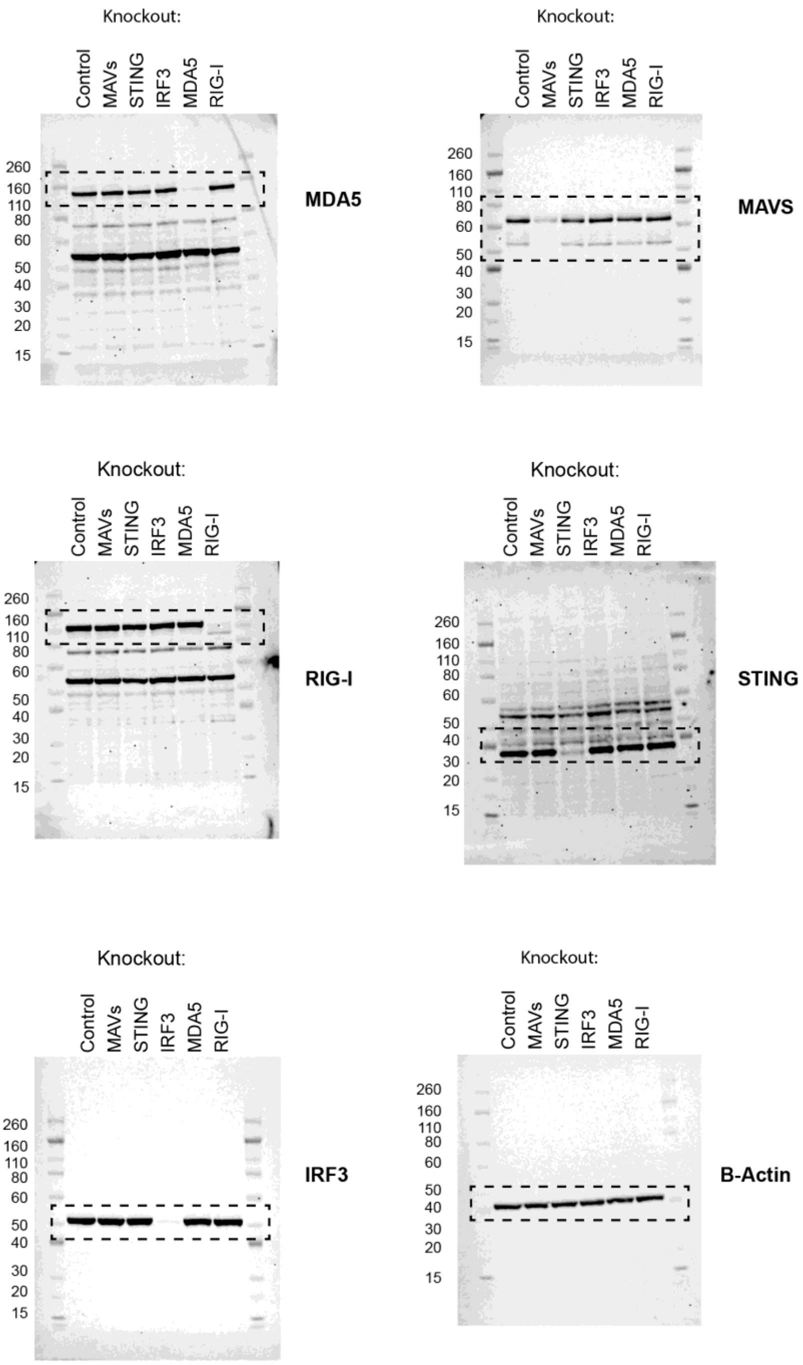

Supplementary Figure 4. Full western blots; corresponding to Figure 3A.
